# Supplementary figures and images for: Differential effects of cotreatment of the antibiotic rifampin with host-directed therapeutics in reducing intracellular Staphylococcus aureus infection
Source: PeerJ. 2020 Nov 10;8:e10330. doi: 10.7717/peerj.10330 (PMC7664464; doi:10.7717/peerj.10330)

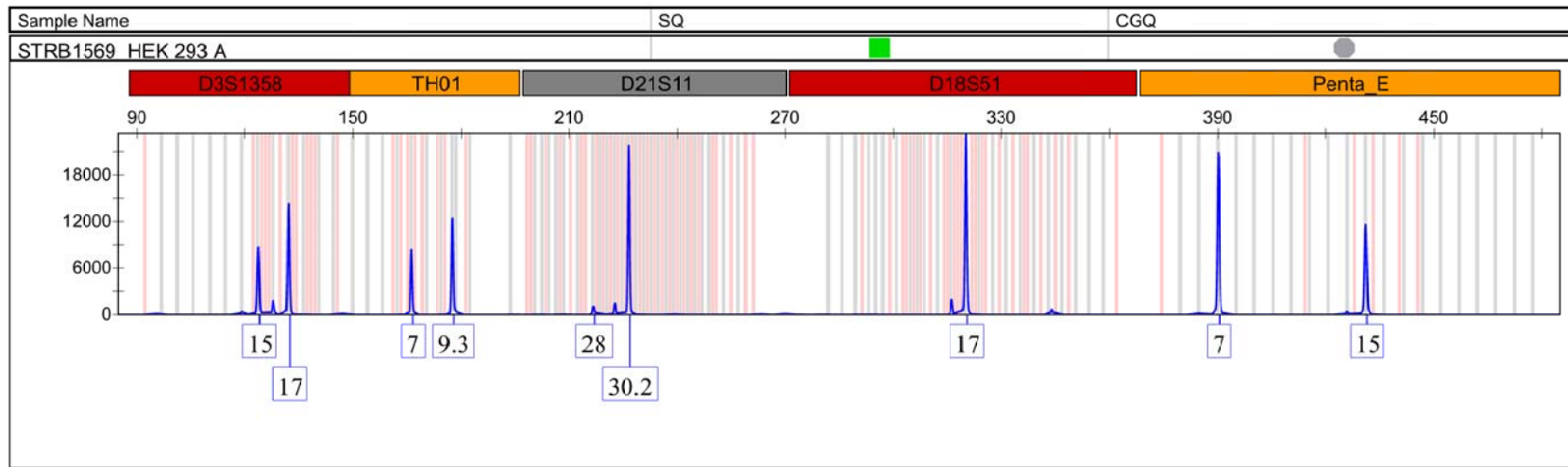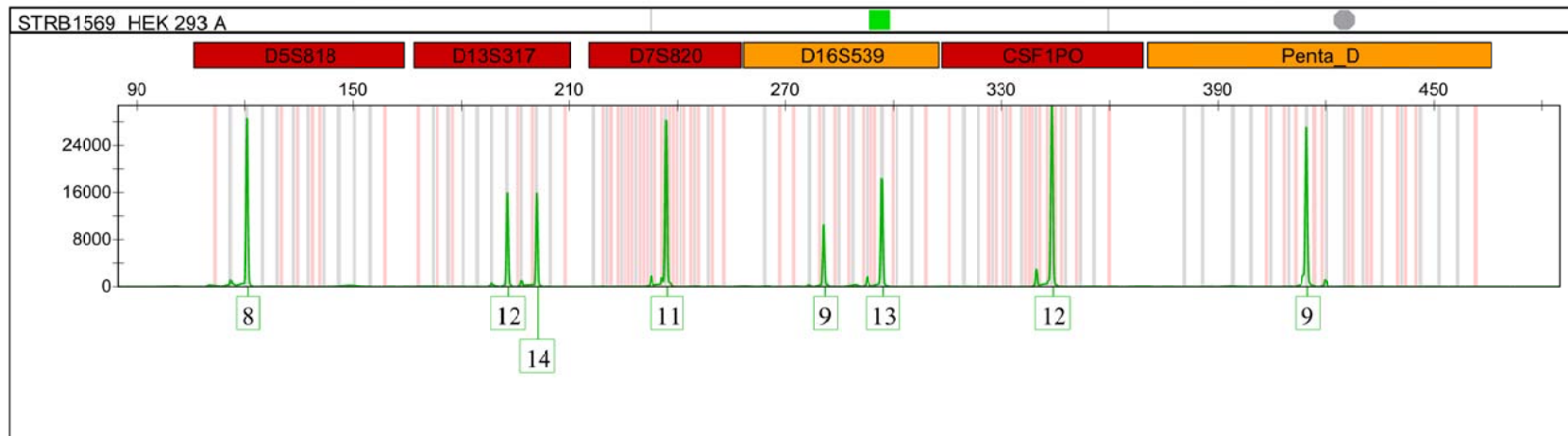

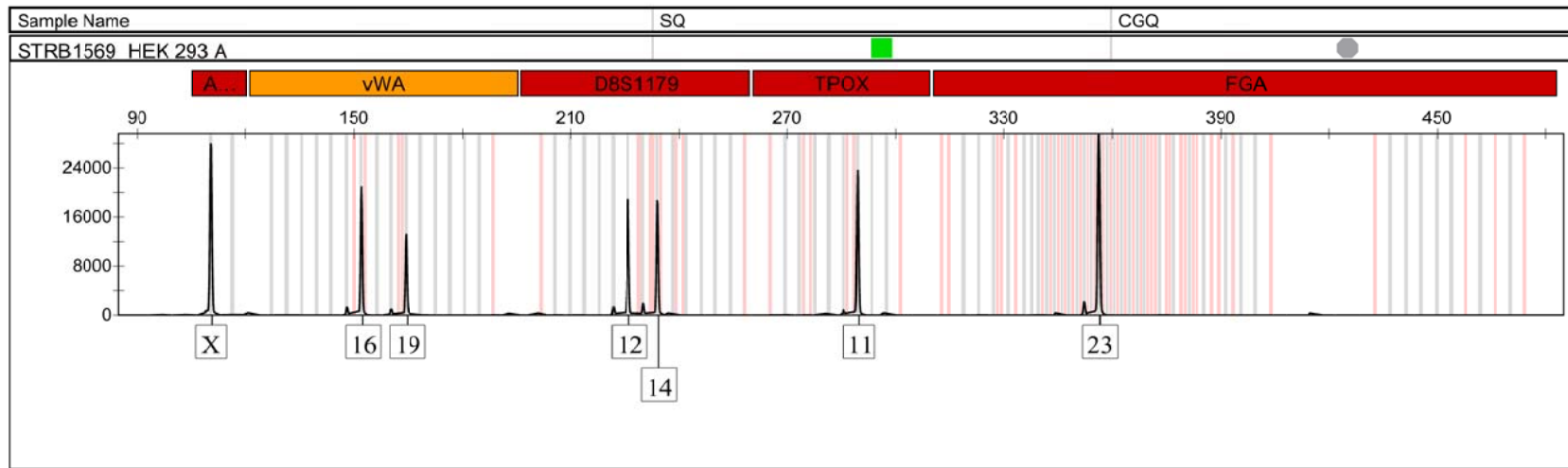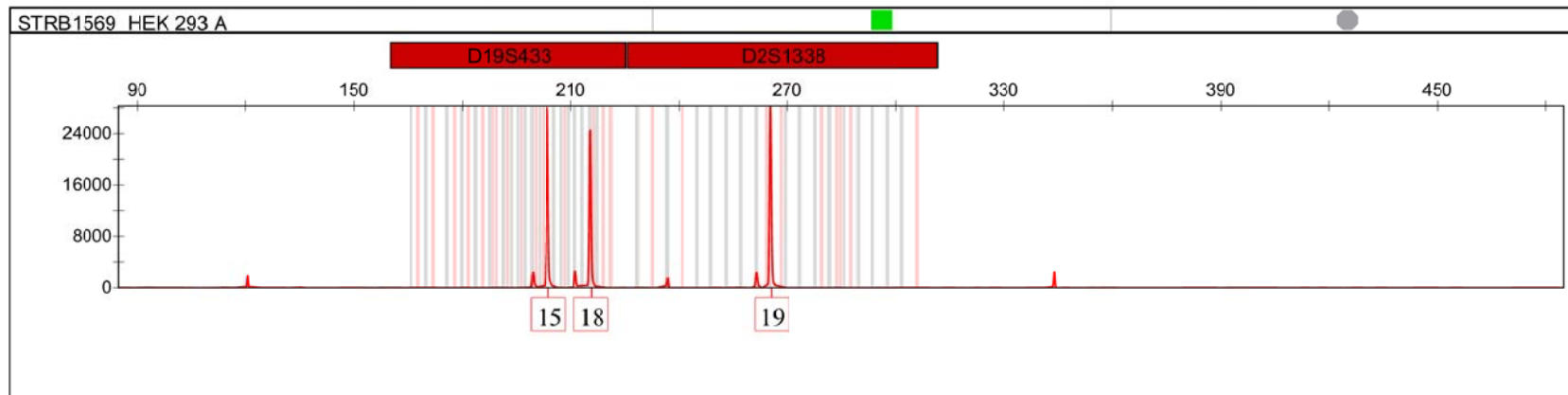

Supplement: Supplemental Information 3 [file peerj-08-10330-s003.pdf]
